# Creep and Recovery Behavior of Continuous Fiber-Reinforced 3DP Composites

**DOI:** 10.3390/polym13101644

**Published:** 2021-05-19

**Authors:** Ans Al Rashid, Muammer Koҫ

**Affiliations:** Division of Sustainable Development, College of Science and Engineering, Hamad Bin Khalifa University, Qatar Foundation, Doha 34110, Qatar; mkoc@hbku.edu.qa

**Keywords:** additive manufacturing, creep and recovery, continuous fiber-reinforced composites, dynamic mechanical analysis, aerospace applications

## Abstract

The commercial availability of 3D printers for continuous fiber-reinforced 3D-printed (CFR3DP) composites has attracted researchers to evaluate the thermomechanical properties of these materials. The improvement of strength through chopped or continuous fiber reinforcements in polymers could provide remarkable results, and its exploration can provide broad applications in several industries. The evaluation of mechanical properties of these materials at elevated temperatures is vital for their utilization in severe operating conditions. This study provides insight into the effect of different fiber reinforcements (Kevlar, fiberglass, and high-strength high-temperature fiberglass) and temperatures on the creep and recovery behavior of CFR3DP Onyx composites. Experimental results were also compared with analytical models, i.e., Burger’s model and Weibull distribution function, for creep and recovery. Results from analytical models agreed well with experimental results for all the materials and temperatures. A significant drop in maximum and residual strains was observed due to the introduction of fibers. However, the creep resistance of all the materials was affected at higher temperatures. Minimum creep strain was observed for Onyx-FG at 120 °C; however, at the same temperature, the minimum residual strain was observed for Onyx-KF. Based on the analytical models and experimental results, the role of fiber reinforcements on the improvement of creep and recovery performance is also discussed.

## 1. Introduction

Additive Manufacturing (AM) (also known as three-dimensional printing (3DP)) processes have been the subject of interest for the past few decades and have attracted several industrial sectors owing to multiple benefits of rapid fabrication, design flexibility, and in low-batch conditions, low cost over traditional manufacturing processes [1]. Based on the working principle, 3DP processes are mainly divided into seven categories: vat polymerization, powder bed fusion, material extrusion, material jetting, binder jetting, directed energy deposition, and sheet lamination processes [2]. Material extrusion processes, more specifically fused filament fabrication (FFF) technique, have been used domestically and in industries due to their cost-effectiveness [3]. 3DP market has made decent progress in fabricating polymer-based components for several industries through extrusion processes [4]. However, physical and mechanical properties of polymers have hindered their utilization due to inferior properties compared to conventional materials such as metals and alloys. Several studies reported improved mechanical properties of polymers through synthetic or natural fibers reinforcement [5,6,7]. However, 3DP of continuous fiber-reinforced polymer (CFRP) composites is still a challenge. Therefore, to utilize freedom of fabrication through 3DP and improve their properties, several efforts have been made on synthesis and 3DP of polymers reinforced with particle or fibrous reinforcement [8,9,10].

Although limited, the commercial availability of 3D printers for continuous fiber-reinforced 3D-printed (CFR3DP) composites has attracted researchers to evaluate properties of these materials for various potential applications [11]. CFRP composites are an important class of materials, as they provide excellent structural and thermomechanical properties. CFR3DP composites offer an opportunity to fabricate high-strength and lightweight components for the automotive, aerospace, and medical industries [12]. Investigation of mechanical behavior of materials primarily included tensile, flexural, fatigue, and creep analysis [6,13]. Besides, several analytical models are also reported in the literature to predict the physical and mechanical properties of the composites. For instance, the recently proposed fractal model can be utilized to investigate the mechanical properties of polymer composites without the need for empirical constants [14]. 

Several studies reported different polymer materials with varying reinforcements for the characterization of CFR3DP composites. Mohammadizadeh et al. [15] reported the tensile, fatigue, and creep analysis of nylon reinforced with carbon fiber (CF), fiberglass (FG), and Kevlar fibers (KF). Kaur et al. [16] investigated 3D-printed octahedral and octet micro trusses produced from polylactic acid (PLA), carbon fiber reinforced PLA, and nylon. Korkees et al. [17] studied the flexural performance of carbon fiber reinforced nylon composites. Their study reported the effect of fiber volume fraction and fiber orientation on flexural properties of nylon–carbon fiber composites.

Onyx is a nylon-based material with improved mechanical properties, owing to the impregnation of carbon microfibers. Bárnik et al. [18,19] studied the effect of infill pattern and density on 3D-printed Onyx samples’ mechanical properties. A rectangular pattern with an even number of layers revealed optimum mechanical performance; however, this infill pattern added the most towards overall volume. Sága et al. [20] also attempted to optimize the 3DP process parameters for enhanced 3D-printed onyx parts. According to their findings, 0°/90° printing orientation provided the highest tensile strength and modulus, whereas, for the ±45° printing pattern, Poisson’s ratio and tensile strain were found optimum. Besides experimental studies, finite element analysis (FEA) tools have also been utilized to study the effect of infill pattern, size of infill geometry, and elastic properties of CFR3DP composites [21,22,23]. Nayak et al. [24] investigated the tensile properties of acrylonitrile butadiene styrene (ABS), PLA, and Onyx reinforced with carbon and Kevlar fibers. FG and high-strength high-temperature FG (HSHT-FG) reinforced onyx composites revealed the best stiffness and loading capacity out of all the materials tested. Zhang et al. [25] examined the wear behavior and mechanical performance of 3D-printed nylon gears, and they performed better than injection molded gears for low-to-medium torque applications. Cuesta et al. [26] studied 3D-printed polymer sheets’ fracture behavior, including Onyx, and proposed a cost-effective technique to evaluate the same for 3D-printed polymers.

The creep phenomenon causes permanent deformation to materials under specific load and is time-dependent. At room temperature, creep deformation is often neglected when applied stresses are significantly below the material’s yield limit, but it is crucial for components requiring higher precisions [27]. Creep becomes even more critical, as it is considered a life-limiting issue under higher loads and elevated temperatures [28]. Understanding the creep and recovery behavior of polymer composites is vital, but not limited to their application in the aerospace and automotive industries. The above-reported literature on polymer composites and the 3DP process mainly focused on optimizing process parameters (infill density, raster orientation, fiber orientation, etc.) or mechanical testing at room temperature. However, investigation on the effect of temperature and time-dependent loading (creep) is merely reported, more specifically for the material reported in this study. Onyx is remarkably strong, exhibits higher strength compared to conventionally used polymers [20]. Furthermore, improvement of strength through continuous fiber reinforcements could provide remarkable results, and its exploration can provide applications in several industries [24,29]. An in-depth examination is vital to evaluate the creep and recovery response of Onyx and its composites.

Dynamic mechanical analysis (DMA) is a sophisticated experimental approach to investigate the effect of temperature and/or frequency/time on the mechanical properties of polymers [30,31]. DMA provides information on storage modulus, loss modulus, and tangent delta, which can also be used for qualitative analysis of composites [30]. The effect of temperature and time-dependent loading on the thermomechanical behavior of CFR3DP Onyx composites is vital to evaluate for their utilization in applications involving elevated temperatures and time-dependent loads. To the best of the authors’ knowledge, there is a gap for a comprehensive study on creep and recovery analysis of CFR3DP composites, specifically for Onyx-based composites [32]. This study provides insight into the effect of different reinforcements (Kevlar, fiberglass, and high-strength high-temperature fiberglass) and temperatures on the creep and recovery behavior of CFR3DP Onyx composites. Experimental results were also compared with analytical models, i.e., Burger’s model and Weibull distribution function, for creep and recovery.

## 2. Materials and Methods

### 2.1. Materials and 3DP Conditions

Onyx (base material) and Kevlar, FG, and HSHT-FG fibers (reinforcements) were obtained from Markforged^®^ company (Watertown, MA, USA) [33]. Onyx itself is a composite material consisting of nylon as a base, reinforced with carbon microfibers. It needs to be stored in a dry box for protection against moisture to avoid property deterioration. Composites with four compositions were 3D printed: pure Onyx and Onyx reinforced with Kevlar fiber, fiberglass, and high strength high-temperature fiberglass (Onyx-KF, Onyx-FG, and Onyx-HSHT-FG, respectively). Markforged^®^ Mark Two printer was used for 3DP of composites specimens. This printer uses two printheads to extrude base material and fibers separately at desired positions.

### 2.2. 3D Printing Process

The specimen geometry of a rectangular plate shape (60 × 12 × 3 mm³) was designed using commercial 3D modeling software, Solidworks^®^ (Dassault Systèmes, Paris, France). Eiger, an exclusive slicing software from Markforged^®^ company (Watertown, MA, USA), was used to define the process parameters and different material compositions. A printing resolution was kept as 0.1 mm for all materials, requiring 30 layers to produce a sample to achieve higher dimensional accuracy. Specimens were printed with 100% infill density, building a solid structure with two wall layers. Printing temperature for onyx and fibers printheads was 275 and 252 °C, respectively. 

3DP process performance strongly depends upon several process parameters. The selection of 3DP process parameters was carefully made to achieve optimum mechanical strength. Continuous fibers placed at 0° to the axial direction provided higher flexural strength and modulus than other orientations for Nylon/Carbon composites [17]. Besides, laminates symmetric about the mid-plane and evenly distributed reinforcement layers provide better mechanical performance than accumulated fiber-reinforcement layers. Finally, higher infill densities offer better structural integrity. With guided knowledge from existing literature, CFR3DP Onyx composites were designed symmetrically about mid-plane and stacking sequence reported in Table 1.

**Figure 1:**
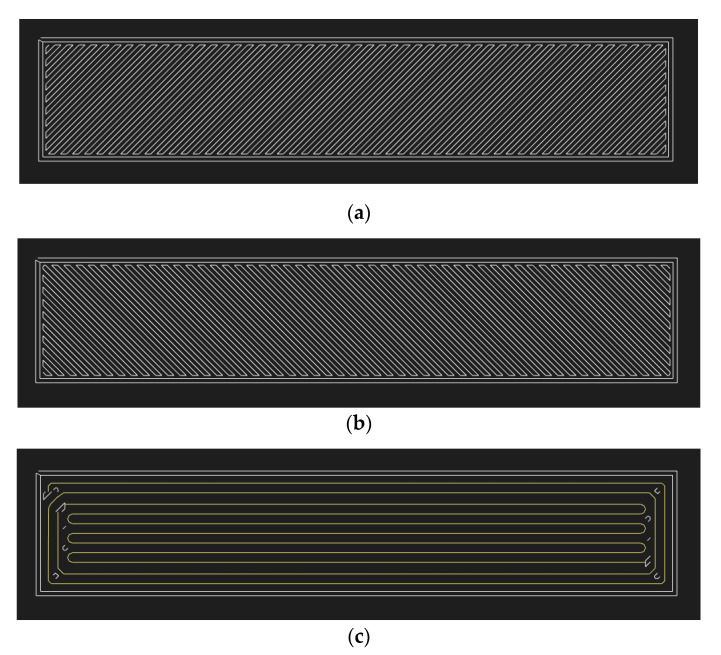
Printing orientation for Onyx and reinforcements. (**a**) Onyx layer with +45° printing orientation; (**b**) Onyx layer with −45° printing orientation; (**c**) Fiber reinforcement layer with hybrid infill

Reinforcement was designed as a hybrid fill type, including two concentric fiber rings and rest with isotropic fibers oriented at 0° to the axial direction. Onyx layers were 3D printed at ±45° printing orientation, as shown in Figure 1. Fiber volume fraction was kept constant at around 38% for all the specimens.

### 2.3. Creep and Recovery Experiments

**Figure 2:**
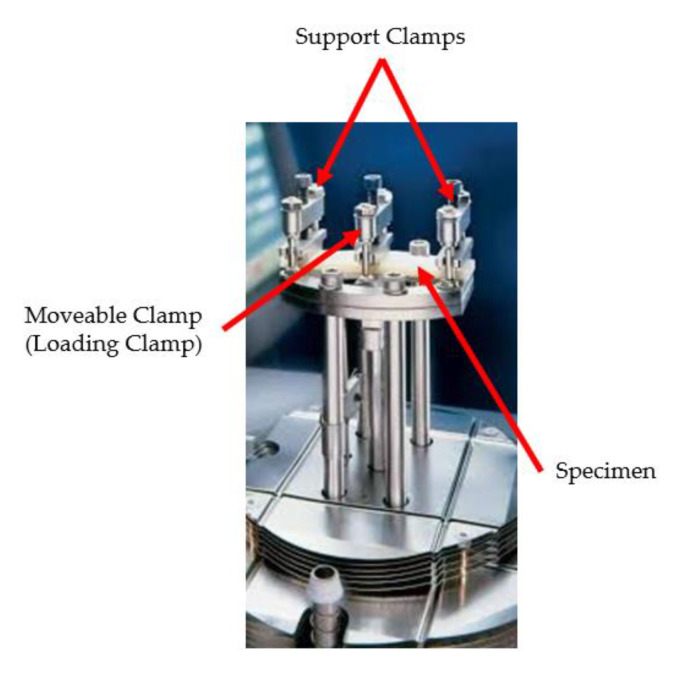
Clamped specimen under dual-cantilever configuration.

The creep and recovery experiments were performed using DMA Q800 (TA Instruments Inc., New Castle, Delaware, US) and under a dual-cantilever configuration following ASTM D4065-20 [34], as shown in Figure 2. Creep and recovery tests were performed at three different temperatures (30, 70, and 120 °C), and the testing temperature was maintained for 5 min before loading the specimens. Constant temperature and stress (of 1 MPa) were applied to the specimens for 60 min to obtain the creep behavior. Finally, a step of 60 min was performed on the removal of the applied load for recovery analysis.

### 2.4. Modeling Viscoelasticity

Creep-time behavior of composite materials is mostly analyzed using Burger’s model, which constitutes Maxwell and Kelvin-Voigt models. Burger’s model proposes the linear summation of three distinct phenomena occurring in linear viscoelastic materials and is expressed as:

(1) $$\epsilon \left(t\right)=\epsilon_{E}+\epsilon_{V}+\epsilon_{P}$$

$\epsilon_{E}$ represents the elastic deformation and is modeled as Maxwell spring element. $\epsilon_{V}$ corresponds to the viscoelastic response of the material and corresponds to the Kelvin-Voigt model. $\epsilon_{P}$ corresponds to the Maxwell dashpot element and represents the permanent deformation.

(2) $$\epsilon \left(t\right)=\frac{\sigma_{o}}{E_{M}}+\frac{\sigma_{o}}{E_{K}}\left[1-\exp\left(-\frac{tE_{K}}{\eta_{K}}\right)\right]+\frac{\sigma_{o}}{\eta_{M}}t$$

where t presents the loading time; $E_{M}$ and $\eta_{M}$ are the modulus of spring and viscosity of dashpot element in Maxwell model, respectively; $E_{K}$ and $\eta_{K}$ are the modulus of spring and viscosity of Kelvin-Voigt elements, respectively; and $\sigma_{o}$ is the applied stress. The ratio $\eta_{K}/E_{K}$ is referred to as the total delay time for 63.2% deformation in the Kelvin model and is denoted as τ.

Elastic deformation restores immediately on removal of applied load, thus strain is a time-dependent function representing the material recovery. The Weibull distribution function is commonly employed to the fit creep recovery process of polymer composites.

(3) $$\epsilon_{R}\left(t\right)=\epsilon_{V}\;\left[exp\;(-{\left(\frac{t-t_{0}}{\eta_{r}}\right)}^{\beta_{r}})\;\right]+\epsilon_{P}$$

where $\epsilon_{V}$ represents the viscoelastic recovery strain, is a function of time and depends upon two critical factors $\beta_{r}$ and $\eta_{r}$ (named shape factor and characteristic life, respectively). $t_{0}$ corresponds to the time at which stress is removed and $\epsilon_{P}$ corresponds to the permanent deformation induced in the material due to viscous flow.

## 3. Results and Discussion

### 3.1. Creep and Recovery Analysis

**Figure 3:**
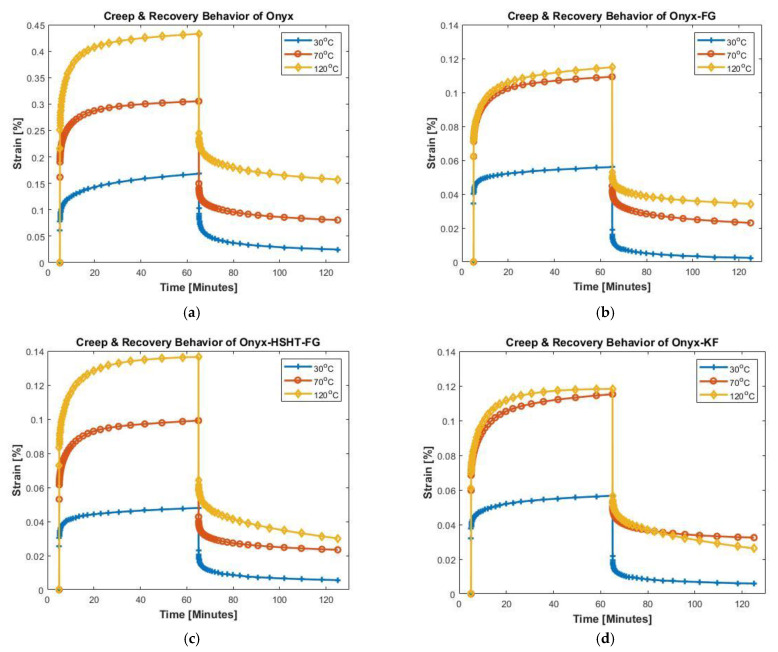
Creep and recovery curves of CFR3DP composites; (**a**) Creep and recovery behavior of Onyx; (**b**) Creep and recovery behavior of Onyx-FG; (**c**) Creep and recovery behavior of Onyx-HSHT-FG; (**d**) Creep and recovery behavior of Onyx-KF.

Figure 3 presents the creep and recovery strain curves for Onyx and CFR Onyx composites as a function of time at different temperatures. The constant stress of 1 MPa was applied for 60 min to all materials for all temperatures selected for experiments, followed by a recovery step of the same duration. Typically, instantaneous elastic deformation, primary creep, and secondary creep constitute the creep behavior of the viscoelastic materials. These three stages of creep are evident from the creep portion of the curves. Creep rupture of the specimens is not apparent due to lower stress levels and shorter creep times. Temperature sensitivity of the materials under observation is also evident as an increased creep and recovery response is observed with increasing temperature for all materials tested due to temperature-activated softening of the polymer material resulting in reduced stiffness. For instance, an increase in temperature from 30 to 70 °C resulted in ~82%, ~95%, ~106%, and ~103% increase in maximum strain for Onyx, Onyx-FG, Onyx-HSHT-FG, and Onyx-KF, respectively.

**Figure 4:**
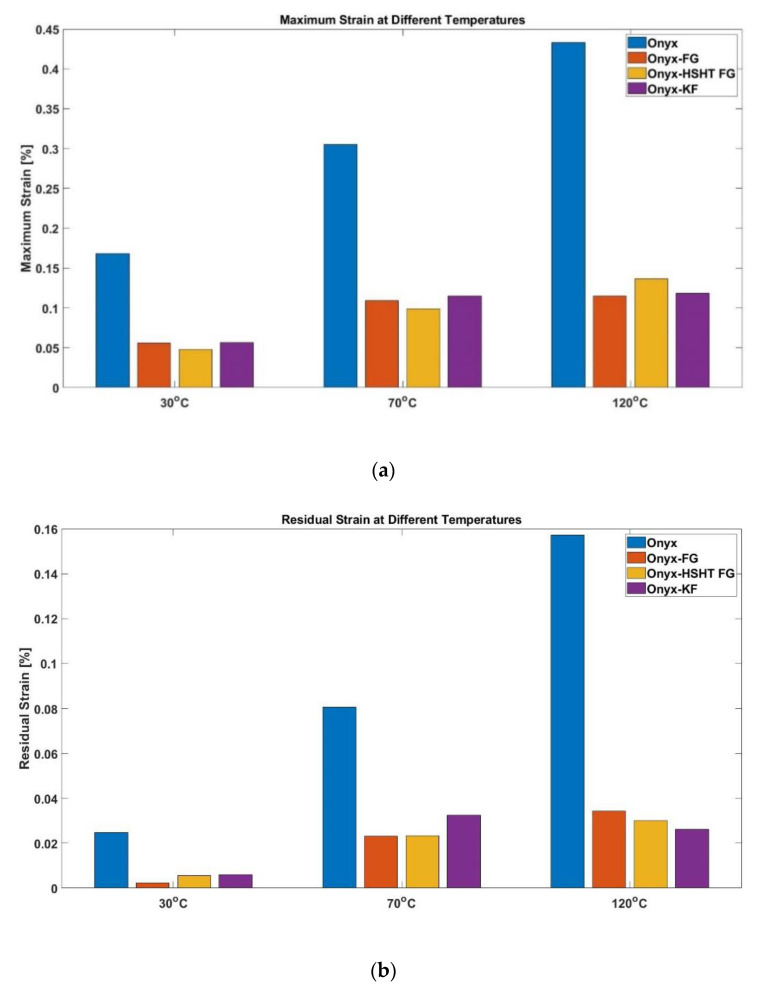
Observed maximum strains for CFR3DP composites at different temperatures; (**a**) Comparison of maximum creep strain; (**b**) Comparison of residual strain.

Similarly, for temperature rise from 30 to 120 °C, maximum strain increased by 157%, 105%, 184%, and 108%, respectively. Interestingly, for Onyx-FG and Onyx-KF, the percentage increase in maximum strain for temperature rise from 70 to 120 °C was significantly lower than the increase observed for 30 to 70 °C temperature rise. However, maximum strain values followed similar behavior for the other two materials, i.e., maximum strain at 120 °C almost doubled the values observed at 70 °C. Bar plots present the maximum strain for all the materials at different temperatures (Figure 4a).

The second portion of the strain vs. time curve presents the recovery behavior of the material. Elastic deformation recovers instantly upon removing the applied load, followed by a delayed strain that shows material recovery and finally an unrecovered strain referred to as a permanent strain. The materials’ recovery behavior is also temperature dependent, as elastic strain is recoverable, but residual strain generally increases with a rise in testing temperature. For instance, in Onyx, 0.0248% residual strain was observed at 30 °C. However, ~225% and ~534% increase in residual strain was observed for temperature rise from 30to 70 °C and 30 to 120 °C, respectively. Bar plots present the residual strains for all the materials at different temperatures (Figure 4b).

The introduction of continuous fiber reinforcements to Onyx significantly improves the creep resistance; however, different fibers contribute differently to the composite material’s creep and recovery behavior. It is also evident in Figure 4 that the impregnation of fiber reinforcements contributed significantly towards the creep behavior of Onyx. The creep strain of composites significantly dropped with fiber reinforcements. The maximum creep strain observed for all CFR Onyx composites at 120 °C is even lower than the maximum creep observed for Onyx at 30 °C. However, the rise in temperature affects the maximum strain in CFR composites. Lower maximum strains were observed at all the temperatures for CFR composites. A similar trend was observed for permanent deformation/residual strain. It is worth mentioning that CFR composites resulted in lower residual strains compared to pure Onyx. Besides, the unrecoverable strain for Onyx also increased for higher temperatures. Therefore, the addition of reinforcements and variation in temperatures remarkably affects the viscoelastic response of the composites.

### 3.2. Creep and Recovery Modeling

**Figure 5:**
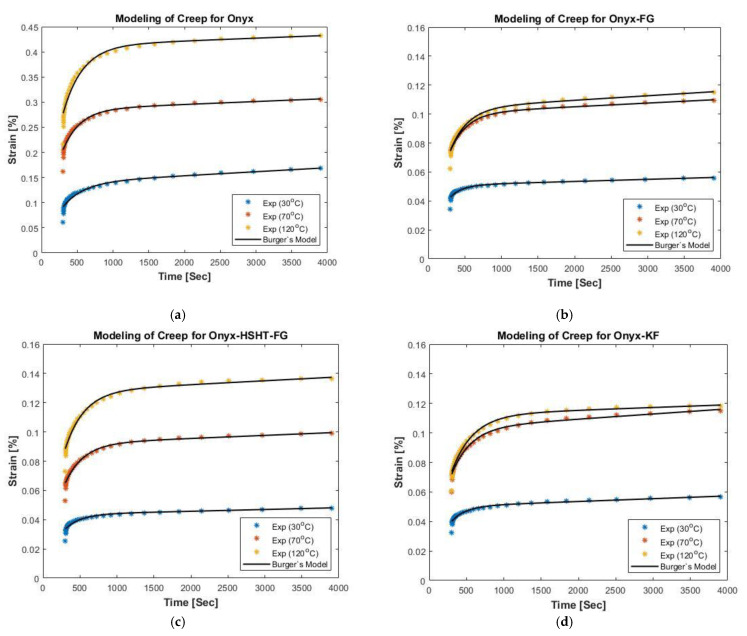
Modeling of creep curves for CFR3DP composites at different temperatures; (**a**) Modeling of creep for Onyx; (**b**) Modeling of creep for Onyx-FG; (**c**) Modeling of creep for Onyx-HSHT-FG; (**d**) Modeling of creep for Onyx-KF.

Burger’s model and Weibull distribution function were used to fit the experimental data obtained for Onyx and CFR composites at different temperatures. Microsoft Excel (Solver tool) was used to fit the experimental data and obtain the parameters of Burger’s model (i.e., $E_{M},\;E_{K},\eta_{M}\;\mathrm{and}\;\eta_{K}$) and Weibull distribution function (i.e., $\beta_{r},\;\eta_{r},\;\epsilon_{V}$ and $\epsilon_{P}$). Figure 5 presents the plotted experimental results for creep and fitted curve with a satisfactory agreement for each temperature level. The evaluated parameters for Burger’s model are reported in Table 2. As mentioned above, $E_{M}$ is the modulus of the spring element in the Maxwell model, where higher $E_{M}$ values correspond to more elastic materials. The higher $E_{M}$ values also refer to higher elasticity and material strength. This component does not appear in the analytical model for creep recovery as elastic strain is instantaneously recovered upon load removal. The $E_{M}$ value increases due to the introduction of continuous fiber reinforcement at a specific temperature, owing to improved strength due to fiber reinforcement. For instance, at 30 °C, $E_{M}$ for CFR composites are higher than pure Onyx. The effect of temperature is also evident on $E_{M}$ values, which are higher at elevated temperatures, owing to the temperature-assisted movement of polymer chains. $\eta_{M}$ represents the dashpot element’s viscosity in the Maxwell model, which contributes significantly to the creep behavior of material and is temperature sensitive. This parameter presents the unrecoverable strain due to permanent deformation in the material. Generally, $\eta_{M}$ values are higher for CFR composites, which corresponds to lower creep rates compared to pure Onyx. Interestingly, $\eta_{M}$ increases with an increase in testing temperature for pure Onyx. In contrast, it decreases for composites with continuous fiber reinforcement. $E_{K}\;\mathrm{and}\;\eta_{K}$ represents the elastic modulus and viscosity of Kelvin-Voigt model elements. The $E_{K}$ value improves with the introduction of continuous fiber reinforcement at a specific temperature. For instance, at 30 °C, $E_{K}$ for CFR composites are higher than pure Onyx. The effect of temperature is also evident on $E_{K}$ values, which is lower at elevated temperatures. $\eta_{K}$ values also revealed higher values for CFR composites than pure Onyx, owing to strong inhibition of polymer relaxation. However, it dropped at higher temperatures.

**Figure 6:**
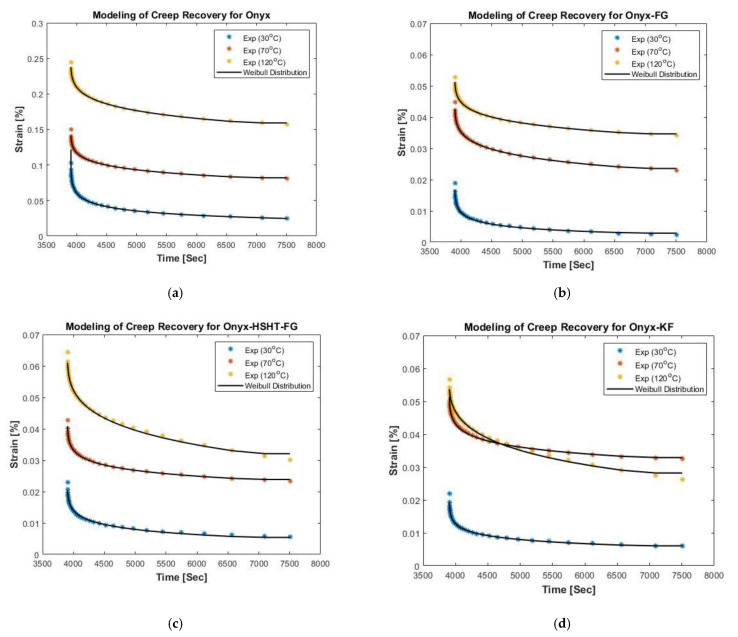
Modeling of creep recovery curves for CFR3DP composites at different temperatures; (**a**) Modeling of creep recovery for Onyx; (**b**) Modeling of creep recovery for Onyx-FG; (**c**) Modeling of creep recovery for Onyx-HSHT-FG; (**d**) Modeling of creep recovery for Onyx-KF.

Figure 6 presents the plotted experimental results for recovery and fitted curve with a satisfactory agreement for each temperature level. The evaluated parameters for the Weibull distribution function are reported in Table 3. $\epsilon_{V}$ and $\epsilon_{P}$ drop significantly due to continuous fiber-reinforcements, which refers to the improved recovery process of these materials. Increased values of viscoelastic strain and permanent strain are also observed at elevated temperatures. However, for CFR composites at 120 °C, these values were still significantly lower than pure Onyx at 30 °C. From Burger’s model and Weibull distribution function, it is concluded that incorporation of continuous fiber as reinforcements to Onyx results in viscosity increase ($\eta_{M}$), which corresponds to reduced molecular chains’ slippage. Consequently, the permanent strain values dropped significantly, resulting in improved recovery behavior of these materials. 

### 3.3. Possible Mechanism in Creep and Recovery

**Figure 7:**
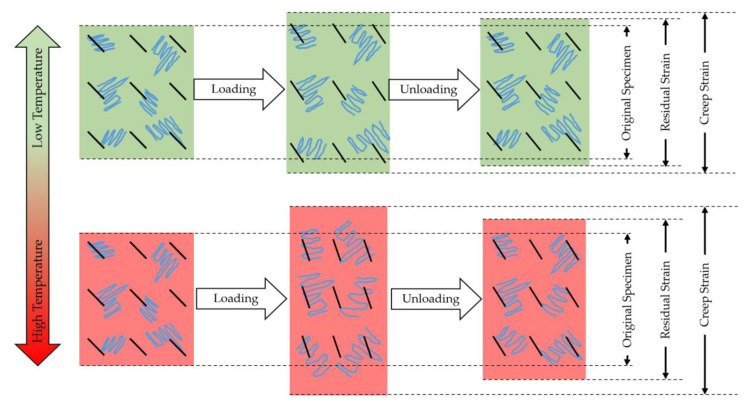
Possible mechanism in creep and recovery (effect of load and temperature on Onyx).

From the results presented, possible mechanisms undergoing in Onyx and its composites are presented in Figure 7. Due to shear forces involved in the extrusion 3DP process, the chopped fibers within the nylon matrix align with the printing orientation during the fabrication process [35,36]. Besides, the continuous fiber-reinforcements are aligned at 90° to the axial direction. Upon loading, the polymer chains, chopped fibers, and continuous fibers become strained. The polymer chains and chopped fibers get aligned with the loading direction. However, the continuous fibers effectively prevent the polymer chains’ slippage and disentanglement, resulting in lower creep deformations. Elevated temperatures assist an increased alignment of polymer chains and chopped fibers with the loading direction; therefore, higher creep deformations are observed at higher temperatures. However, continuous fibers play a vital role in hindering the significant polymer chains stretching and sliding of polymer molecules [37].

When the applied load is removed, elastic deformation is instantly recovered, and composite materials start the recovery process [38]. Besides, the relaxation of polymer chains also starts. At lower temperatures, the polymer chains can withstand higher loads, restraining the permanent deformation. However, at higher temperatures, the polymer chains can relatively undergo higher polymer molecules stretching, resulting in higher permanent deformations (residual strains). Continuous fibers provide higher elasticity and strength to the base material, leading to easier recovery to the initial state. The recoverable strain is highly dependent on the type of fiber reinforcement and its interfacial adhesion with the polymer. 

Based on the discussion and coherence with the presented experimental results, the creep and recovery behavior of chopped fiber-reinforced composites is strongly dependent on the applied load and temperature. Continuous reinforcement significantly reduces the creep strain and residual permanent deformation, but it depends on the type of reinforcement and its interfacial bonding with the matrix (Onyx).

## 4. Conclusions

This study provides insight into the effect of different reinforcements (Kevlar, fiberglass, and high-strength high-temperature fiberglass) and temperature levels on the creep and recovery behavior of 3D-printed CFR3DP Onyx composites. Experimental results were also compared with analytical models, i.e., Burger’s model and Weibull distribution function, for creep and recovery. Results from analytical models agreed well with experimental results for all the materials and temperatures. A significant drop in maximum and residual strains was observed due to the introduction of fibers; however, the creep resistance of all the materials was affected at higher temperatures. From experimental results, it can be concluded that the addition of reinforcements and variation in temperatures remarkably affects the composites’ viscoelastic response. Lower creep and residual strains were observed for CFR Onyx composites, but both were affected by higher temperatures. Minimum creep strain was observed for Onyx-FG at 120 °C; however, at the same temperature, the minimum residual strain was observed for Onyx-KF. The experimental creep results and fitted curve (Burger’s Model and Weibull distribution function) had a satisfactory agreement for each temperature level. The parameters obtained for both analytical models and the associated microstructural behavior of materials resulted in improved properties. The improved elastic moduli and viscosities due to continuous fibers’ presence resulted in strong inhibition of molecular relaxation and reduced molecular chains’ slippage, consequently enhancing creep resistance and recovery behavior.

This study presented the creep and recovery performance of selected materials at elevated temperatures. All specimens were fabricated under similar process conditions. As reported in the literature, the tested materials are expected to provide optimum performance under these process parameters; however, future studies may investigate further this effect, such as variation in infill density, raster orientation, fiber orientation, etc., for each material. The analytical models presented here successfully predicted the creep and recovery behavior of materials. Insight into the parameters obtained from these models is also presented and linked with physical mechanisms causing creep and recovery. However, future studies will also focus on implementing temperature-dependent models for prediction at temperatures other than considered during tested. 

## Figures and Tables

**Table 1:** Specimen Specifications and 3DP Parameters.

**Specimen Dimensions**	60 × 12 × 3 mm³ (L × W × T)
**Printing Resolution**	0.10 mm
**Fill Pattern**	Solid Fill
**Fill Density**	100%
**Wall Layers**	2
**Printing Orientation (Onyx)**	±45°
**Fiber Volume Fraction**	~38%
**Reinforcement Fill Type**	Hybrid (Concentric + Isotropic)
**Concentric Rings**	2
**Isotropic Reinforcement Angle**	0ᴼ
**Stacking Sequence**	[4O/4F/2O/4F/1O]s
**Printing Temperature (Onyx)**	275 °C
**Printing Temperature (Reinforcements)**	252 °C

**Table 2:** Simulated parameters of the Burger’s model.

Temperature	Material	$E_{M}\;\left(MPa\right)$	$E_{K}$ (MPa)	$\eta_{K}\;\left(MPa\cdot s\right)\;\times \;10^{3}$	$\eta_{M}$ $\left(MPa\cdot s\right)\;\times \;10^{3}$
30 °C	Onyx	342,409	7.26	2.09	126
	Onyx-FG	342,457	19.79	3.33	687
	Onyx-HSHT-FG	342,483	23.09	4.80	821
	Onyx-KF	342,457	20.10	3.84	528
70 °C	Onyx	342,449	3.52	0.84	171
	Onyx-FG	342,500	10.00	2.27	393
	Onyx-HSHT-FG	342,511	10.94	2.71	479
	Onyx-KF	342,439	9.77	2.50	287
120 °C	Onyx	342,534	2.43	0.66	181
	Onyx-FG	342,530	9.68	2.33	321
	Onyx-HSHT-FG	342,532	7.87	2.03	379
	Onyx-KF	342,470	8.95	2.54	543

**Table 3:** Simulated parameters of the Weibull distribution function.

Temperature	Material	*Ԑ* *_V_* *(%)*	*η_r_ (s)*	*β_r_*	*Ԑ* *_P_* *(%)*
30 °C	Onyx	0.113	250.43	0.253	0.009
	Onyx-FG	0.023	201.90	0.252	0
	Onyx-HSHT-FG	0.024	955.99	0.298	0
	Onyx-KF	0.026	520.63	0.213	0
70 °C	Onyx	0.124	3971.48	0.248	0.035
	Onyx-FG	0.047	15,062.15	0.249	0
	Onyx-HSHT-FG	0.039	3505.37	0.202	0.009
	Onyx-KF	0.042	3536.11	0.206	0.017
120 °C	Onyx	0.171	8371.30	0.293	0.081
	Onyx-FG	0.041	15,483.37	0.252	0.014
	Onyx-HSHT-FG	0.064	10,196.39	0.357	0
	Onyx-KF	0.044	4210.91	0.435	0.011

## Data Availability

Raw data will be available upon request to the corresponding author.

## References

[1] Ngo T.D., Kashani A., Imbalzano G., Nguyen K.T., Hui D. (2018). Additive manufacturing (3D printing): A review of materials, methods, applications and challenges. Compos. Part B Eng..

[2] Al Rashid A., Khan S.A., Al-Ghamdi S.G., Koç M. (2020). Additive manufacturing: Technology, applications, markets, and opportunities for the built environment. Autom. Constr..

[3] Tofail S.A., Koumoulos E.P., Bandyopadhyay A., Bose S., O’Donoghue L., Charitidis C. (2018). Additive manufacturing: Scientific and technological challenges, market uptake and opportunities. Mater. Today.

[4] González-Henríquez C.M., Sarabia-Vallejos M.A., Rodriguez-Hernandez J. (2019). Polymers for additive manufacturing and 4D-printing: Materials, methodologies, and biomedical applications. Prog. Polym. Sci..

[5] Sánchez D.M., de la Mata M., Delgado F.J., Casal V., Molina S.I. (2020). Development of carbon fiber acrylonitrile styrene acrylate composite for large format additive manufacturing. Mater. Des..

[6] Khalid M.Y., Nasir M.A., Ali A., Al Rashid A., Khan M.R. (2020). Experimental and numerical characterization of tensile property of jute/carbon fabric reinforced epoxy hybrid composites. SN Appl. Sci..

[7] Karakoç A., Rastogi V.K., Isoaho T., Tardy B., Paltakari J., Rojas O.J. (2020). Comparative Screening of the Structural and Thermomechanical Properties of FDM Filaments Comprising Thermoplastics Loaded with Cellulose, Carbon and Glass Fibers. Materials.

[8] Malas A., Isakov D., Couling K., Gibbons G.J. (2019). Fabrication of High Permittivity Resin Composite for Vat Photopolymerization 3D Printing: Morphology, Thermal, Dynamic Mechanical and Dielectric Properties. Materials.

[9] Al Rashid A., Khalid M.Y., Imran R., Ali U., Koc M. (2020). Utilization of Banana Fiber-Reinforced Hybrid Composites in the Sports Industry. Materials.

[10] Spinelli G., Lamberti P., Tucci V., Kotsilkova R., Ivanov E., Menseidov D., Naddeo C., Romano V., Guadagno L., Adami R. (2019). Nanocarbon/Poly(Lactic) Acid for 3D Printing: Effect of Fillers Content on Electromagnetic and Thermal Properties. Materials.

[11] Van de Werken N., Tekinalp H., Khanbolouki P., Ozcan S., Williams A., Tehrani M. (2020). Additively manufactured carbon fiber-reinforced composites: State of the art and perspective. Addit. Manuf..

[12] Blok L., Longana M., Yu H., Woods B. (2018). An investigation into 3D printing of fibre reinforced thermoplastic composites. Addit. Manuf..

[13] Al Rashid A., Imran R., Khalid M.Y. (2020). Determination of opening stresses for railway steel under low cycle fatigue using digital image correlation. Theor. Appl. Fract. Mech..

[14] Xiao B., Huang Q., Chen H., Chen X., Long G. (2021). A fractal model for capillary flow through a single tortuous capillary with roughened surfaces in fibrous porous media. Fractals.

[15] Mohammadizadeh M., Imeri A., Fidan I., Elkelany M. (2019). 3D printed fiber reinforced polymer composites—Structural analysis. Compos. Part B Eng..

[16] Kaur M., Yun T.G., Han S.M., Thomas E.L., Kim W.S. (2017). 3D printed stretching-dominated micro-trusses. Mater. Des..

[17] Korkees F., Allenby J., Dorrington P. (2020). 3D printing of composites: Design parameters and flexural performance. Rapid Prototyp. J..

[18] Bárnik F., Vaško M., Handrik M., Dorčiak F., Majko J. (2019). Comparing mechanical properties of composites structures on Onyx base with different density and shape of fill. Transp. Res. Procedia.

[19] Bárnik F., Vaško M., Sága M., Handrik M., Sapietová A. (2019). Mechanical properties of structures produced by 3D printing from composite materials. MATEC Web Conf..

[20] Sága M., Bárnik F., Vaško M., Handrik M., Kopas P. (2020). Identification of Physical Characteristic of Composite Materials Produced by Additive Technology from Perspective of Selected Mechanical Properties. Acta Phys. Pol. A.

[21] Dorčiak F., Vaško M., Handrik M., Bárnik F., Majko J. (2019). Tensile test for specimen with different size and shape of inner structures created by 3D printing. Transp. Res. Procedia.

[22] Sága M., Majko J., Handrik M., Vaško M.M., Sapietová A. (2020). Proposal of Physical Model for Damage Simulation of Composite Structures Produced by 3D Printing. Acta Phys. Pol. A.

[23] Yang P., Hu N., Guo X., Dong L., Chen Y., Guo Z. (2020). An ultra-simple universal model for the effective elastic properties of isotropic 3D closed-cell porous materials. Compos. Struct..

[24] Nayak S.M., Shetty P.B., Mishra R.K., Reddy S., Viraj G.R. (2019). Failure Analysis of Additive Manufactured Fiber-Reinforced Thermoplastics. J. Fail. Anal. Prev..

[25] Zhang Y., Purssell C., Mao K., Leigh S. (2020). A physical investigation of wear and thermal characteristics of 3D printed nylon spur gears. Tribol. Int..

[26] Cuesta I., Martinez-Pañeda E., Díaz A., Alegre J. (2019). The Essential Work of Fracture parameters for 3D printed polymer sheets. Mater. Des..

[27] Liu C., Liu P., Zhao Z., Northwood D.O. (2001). Room temperature creep of a high strength steel. Mater. Des..

[28] Wilshire B., Cocks A.C.F., Ponter A.R.S. (1991). Microscopic Models and Macroscopic Constitutive Laws for High Temperature Creep and Creep Fracture of Metallic and Ceramic Materials. Mechanics of Creep Brittle Materials 2.

[29] Beaulieu A., Linden A.Z., Phillips J., Arroyo L.G., Koenig J., Monteith G. (2019). Various 3D printed materials mimic bone ultrasonographically: 3D printed models of the equine cervical articular process joints as a simulator for ultrasound guided intra-articular injections. PLoS ONE.

[30] Xu X., Koomson C., Doddamani M., Behera R.K., Gupta N. (2019). Extracting elastic modulus at different strain rates and temperatures from dynamic mechanical analysis data: A study on nanocomposites. Compos. Part. B Eng..

[31] Zeltmann S.E., Prakash K.A., Doddamani M., Gupta N. (2017). Prediction of modulus at various strain rates from dynamic mechanical analysis data for polymer matrix composites. Compos. Part. B Eng..

[32] Kabir S.M.F., Mathur K., Seyam A.-F.M. (2020). A critical review on 3D printed continuous fiber-reinforced composites: History, mechanism, materials and properties. Compos. Struct..

[33] Markforged. https://markforged.com/.

[34] ASTM International (2008). ASTM D4065-20—Standard Practice for Plastics: Dynamic Mechanical Properties.

[35] Zhong W., Li F., Zhang Z., Song L., Li Z. (2001). Short fiber reinforced composites for fused deposition modeling. Mater. Sci. Eng. A.

[36] Brenken B., Barocio E., Favaloro A., Kunc V., Pipes R.B. (2018). Fused filament fabrication of fiber-reinforced polymers: A review. Addit. Manuf..

[37] Dai Z., Gao Y., Liu L., Pötschke P., Yang J., Zhang Z. (2013). Creep-resistant behavior of MWCNT-polycarbonate melt spun nanocomposite fibers at elevated temperature. Polymer.

[38] Jia Y., Jiang Z., Peng J., Gong X., Zhang Z. (2012). Resistance to time-dependent deformation of polystyrene/carbon nanotube composites under cyclic tension. Compos. Part. A Appl. Sci. Manuf..

